# The Microbiome of *Seriola lalandi* of Wild and Aquaculture Origin Reveals Differences in Composition and Potential Function

**DOI:** 10.3389/fmicb.2017.01844

**Published:** 2017-09-26

**Authors:** Carolina Ramírez, Jaime Romero

**Affiliations:** ^1^Laboratorio de Biotecnología de los Alimentos, Instituto de Nutrición y Tecnología de los Alimentos, Universidad de Chile, Santiago, Chile; ^2^Doctorado en Acuicultura, Programa Cooperativo Universidad de Chile, Universidad Católica del Norte, Pontificia Universidad Católica de Valparaíso, Santiago, Chile

**Keywords:** microbiota, high-throughput sequencing, yellowtail, *Seriola*

## Abstract

*Seriola lalandi* is an economically important species that is globally distributed in temperate and subtropical marine waters. Aquaculture production of this species has had problems associated with intensive fish farming, such as disease outbreaks or nutritional deficiencies causing high mortalities. Intestinal microbiota has been involved in many processes that benefit the host, such as disease control, stimulation of the immune response, and the promotion of nutrient metabolism, among others. However, little is known about the potential functionality of the microbiota and the differences in the composition between wild and aquacultured fish. Here, we assayed the V4-region of the 16S rRNA gene using high-throughput sequencing. Our results showed that there are significant differences between *S. lalandi* of wild and aquaculture origin (ANOSIM and PERMANOVA, *P* < 0.05). At the genus level, a total of 13 genera were differentially represented between the two groups, all of which have been described as beneficial microorganisms that have an antagonistic effect against pathogenic bacteria, improve immunological parameters and growth performance, and contribute to nutrition. Additionally, the changes in the presumptive functions of the intestinal microbiota of yellowtail were examined by predicting the metagenomes using PICRUSt. The most abundant functional categories were those corresponding to the metabolism of cofactors and vitamins, amino acid metabolism and carbohydrate metabolism, revealing differences in the contribution of the microbiota depending on the origin of the animals. To our knowledge, this is the first study to characterize and compare the intestinal microbiota of *S. lalandi* of wild and aquaculture origin using high-throughput sequencing.

## Introduction

*Seriola lalandi* (yellowtail kingfish) is a marine, pelagic, and carnivorous fish found globally in subtropical and temperate waters of the Pacific and Indian Oceans (Fowler et al., 2003). This species is important for aquaculture in Australia (Hutson et al., 2007), New Zealand (Moran et al., 2008), Japan (Nakada, 2002), and Chile, where it is part of the Chilean aquaculture diversification Program (Programa de Diversificación de la Acuicultura Chilena, PDACH). This species has excellent attributes that promote its cultivation, including high rates of growth and market acceptance (Poortenaar et al., 2001). However, disease outbreaks have increased with the intensification of aquaculture, especially during the early stages of fish development; an example of this problem in larval culture is vibriosis (Toranzo et al., 2005). Nutritional deficiencies are another important point to consider among the causes of mortalities in aquacultured fish species (Chen et al., 2007). Therefore, protecting cultured fish from diseases is essential for the sustainability of the aquaculture industry. The control of bacterial diseases in fish culture is traditionally countered by the use of antibiotics. Several authors have warned about the negative effects of the excessive use of antibiotics, which can lead to the selection of antibiotic-resistant bacteria (Dang et al., 2009; Romero et al., 2012). Therefore, the manipulation of the intestinal microbiota through the supplementation of beneficial microbes is presented as an alternative to overcome the adverse effects of antibiotics and drugs (Nayak, 2010).

The intestinal microbiota is considered important for the host, since the microorganisms that reside in the digestive tract may influence many biological processes that generate benefits to the host. Examples of which include providing enzymes to complement the digestion processes; supplying vitamins to enhance nutrition; preventing colonization by pathogens, competing for nutrients and adhesion sites; producing antimicrobial substances; and modulating the host immune system (Verschuere et al., 2000; Rawls et al., 2004; Chabrillón et al., 2005; Ringø et al., 2006; Hovda et al., 2007). This microbial community can be subcategorized into two groups. One group simply passes through the lumen with food or digesta—the allochthonous microbiota, and the other group is potentially resident and intimately associated with host tissues—the autochthonous microbiota (Gajardo et al., 2016; Tarnecki et al., 2017). The use of beneficial bacteria in aquaculture has been recently revised by Akhter et al. (2015). Several studies reported that indigenous bacteria are more beneficial than bacteria isolated from other sources (Nayak, 2010; Mills et al., 2011). This may be due at least in part to the specificity in colonization by the host-strain relationship (Ying et al., 2007).

Sullam et al. (2012) identified a significant association between the intestinal microbiota composition and fish taxonomy, suggesting a potential co-evolution of fishes and their gut microbiota. However, there are few studies that report the impact of fish microbiota under aquaculture conditions. In this context, the aim of this study is to evaluate possible differences in the microbiota associated to the intestinal contents of *S. lalandi* of wild and aquaculture origin using next generation sequencing (NGS) and to explore the potential importance of these differences to the host.

## Materials and methods

### Sample collection

Intestinal content samples were collected from aquaculture and wild *S. lalandi* and were immediately stored at −20°C. Yellowtail kingfish specimens from an aquaculture facility were collected from the Universidad Católica del Norte (Coquimbo, Chile; latitude S 29.966; longitude W 71.751), which were reared in a land based recirculating aquaculture system (RAS), using conditions as described by Aguilera et al. (2013) and diet described in Table S4. Five animals (roughly 3–5 kg) without deformities or apparent illnesses were used for feces collection. The intestinal contents were obtained with the help of a catheter and performing soft massage on the abdomen of fish that had been anesthetized with DOLICAL® 80% (Centrovet, Santiago, Chile). Yellowtail kingfish specimens from the wild environment were collected from latitude S 30.104; longitude W 71.377 to latitude S 30.302; longitude W 70.608, during December 2015. After caught, fish were kept in ice until processing, and five animals (roughly 3–4 kg) were included in this study. The intestinal contents were obtained as described above. This study followed the recommendations of the Guide for the Care and Use of Laboratory Animals of the National Institutes of Health. The Committee on the Ethics of Animal Experiments of the INTA Universidad de Chile approved the protocol.

### DNA extraction and sequencing

DNA was extracted from the intestinal content samples (0.25 g) using the MO BIO PowerFecal®DNA Isolation Kit (MO BIO Laboratories, Carlsbad, CA, USA) according to the manufacturer's protocol. DNA concentrations were measured fluorometrically using the High Sensitivity (HS) kit on the Qubit Fluorometer 3.0 (Invitrogen Co., Carlsbad, USA). The V4 region of the 16S rRNA gene was amplified by the fusion primer method using the primers 515F and 806R described by Caporaso et al. (2011). Variable region 4 was selected because of its high coverage, low error rate and minimum loss of taxonomic resolution (Kuczynski et al., 2012; Lokesh and Kiron, 2016). In addition, the resulting amplicons are of suitable length for use with the Ion Torrent™ sequencing platform (Life Technologies). All PCR reactions were performed as described by Lokesh and Kiron (2016), except volume reaction was 25 μL; each reaction mixture containing 22.5 μL of Platinium PCR SuperMix High Fidelity (Life Technologies), 0.25 μL of primer mix (200 nM) and 2.25 μL of the template DNA (~20 ng). A negative PCR control without the DNA template was also included in the run. The PCR conditions included an initial denaturation at 94°C for 5 min, followed by 35 cycles of denaturation at 94°C for 30 s, annealing at 56°C for 30 s, and extension at 68°C for 45 s. After the cycling procedure, the amplicons from each sample were pooled and run on a 1% agarose gel. Subsequently, the amplicons were purified with the QIAquick PCR Purification kit (Qiagen, Valencia, CA). DNA sequencing was performed via Ion Torrent Personal Genome Machine system (Life Technologies, California) using a 318 chip at the facilities of the University of Plymouth Enterprise Ltd.

### Bioinformatics analyses

Sequencing reads of the 16S rRNA gene were processed and analyzed using UPARSE (Edgar, 2013) and QIIME (Caporaso et al., 2010), as described previously by Ramírez and Romero (2017), except that the sequences were trimmed to 270 bp. Sequences assigned to chloroplast and unclassified at the kingdom level, were removed. OTUs containing < 10 sequences were also removed from the dataset. The analyses of diversity indexes included Good's coverage, alpha diversity indexes comprising community diversity (Simpson and Shannon index), richness (Chao-1) and phylogeny-based metrics (PD Whole Tree), which were calculated using QIIME. Principal coordinates analysis (PCoA) was used to evaluate the Beta diversity obtained by unweighted UniFrac and weighted UniFrac analyses, using the “beta_diversity.py” QIIME script. EMPeror was used to visualize the PCoA plots from the unweighted and weighted UniFrac metrics. The inferred metagenomics and predicted functional analysis was performed using PICRUSt as described previously by Ramírez and Romero (2017), and it included all the readings (325,039 from wild; 314,323 from aquaculture). The accuracy of the predictions of the metagenomes was assessed by computing the NSTI (Nearest Sequenced Taxon Index), which is an index that indicates the relationship of the microbes in a particular sample to the bacterial genomes in a database (Table S3). The associated metabolic pathways were deciphered by employing HUMAnN2 (The HMP Unified Metabolic Analysis Network) with the default settings.

### Statistical analyses

Shannon Diversity index, Simpson index, richness and PD Whole Tree were included to examine the differences in alpha diversity between the wild and aquacultured yellowtail kingfish. The normality was tested with Shapiro and Wilk test (Shapiro and Wilk, 1965). The comparisons were done with student *t*-test or Mann-Whitney test when distributions of data were normal or non-normal, respectively. The calculations were made in GraphPad Prism 6 (GraphPad Software, Inc., La Jolla, CA, USA) with *P* < 0.05 considered statistically significant. The UniFrac distance matrices were analyzed by Analysis of similarities (ANOSIM) and Permutation multivariate analysis of variance (PERMANOVA) with 9999 permutations. For this purpose, the dissimilarity matrix for the unweighted and weighted UniFrac were analyzed in QIIME, with *P* < 0.05 considered statistically significant. To identify differentially abundant OTUs between two origins, the DESeq2 software package was employed considering *P* < 0.05 (Love et al., 2014). The *t*-test was used to evaluate bacterial functional pathways that were differentially abundant in intestinal microbiota of wild yellowtail and aquaculture yellowtail kingfish. All *P*-values were corrected with the Benjamini–Hochberg false discovery rate method.

### Data deposition

Raw sequences from 16S rRNA gene profiling are accessible through the SRA study accession numbers: SRS2110405, SRS2110403, SRS2110404, SRS2110402, SRS2110401, SRS2110400, SRS2110398, SRS2110397, SRS2110399, SRS2110396.

## Results

### Sequencing depth

Intestinal contents were collected from wild (*n* = 5) and aquacultured (*n* = 5) *S. lalandi*. The microbiota composition was analyzed using barcoded sequencing of the V4 region of the 16S rRNA gene. After filtering as described above, 632,079 reads remained; 329,314 reads from wild *S. lalandi* with an average of 65,863 ± 10,706 reads per individual sample. And 302,765 reads from aquacultured *S. lalandi* with an average of 60,553 ± 5,663 reads per individual sample. These 270 bp sequences were assigned to 683 operational taxonomic units (OTUs) based on 97% similarity using QIIME. Good's coverage estimators for all samples were >0.99, indicating that sufficient sequencing coverage was achieved and that the OTUs detected in the samples are representative of the sampled population (Table S1). Rarefaction curves based on alpha diversity metrics (Chao1 and PD whole tree) reached the saturation phase in wild and aquaculture yellowtail at 30,000 sequence readings (Supplementary Figures S1, S2).

### Diversity analysis of microbiota of wild and aquacultured yellowtail

**Figure 1:**
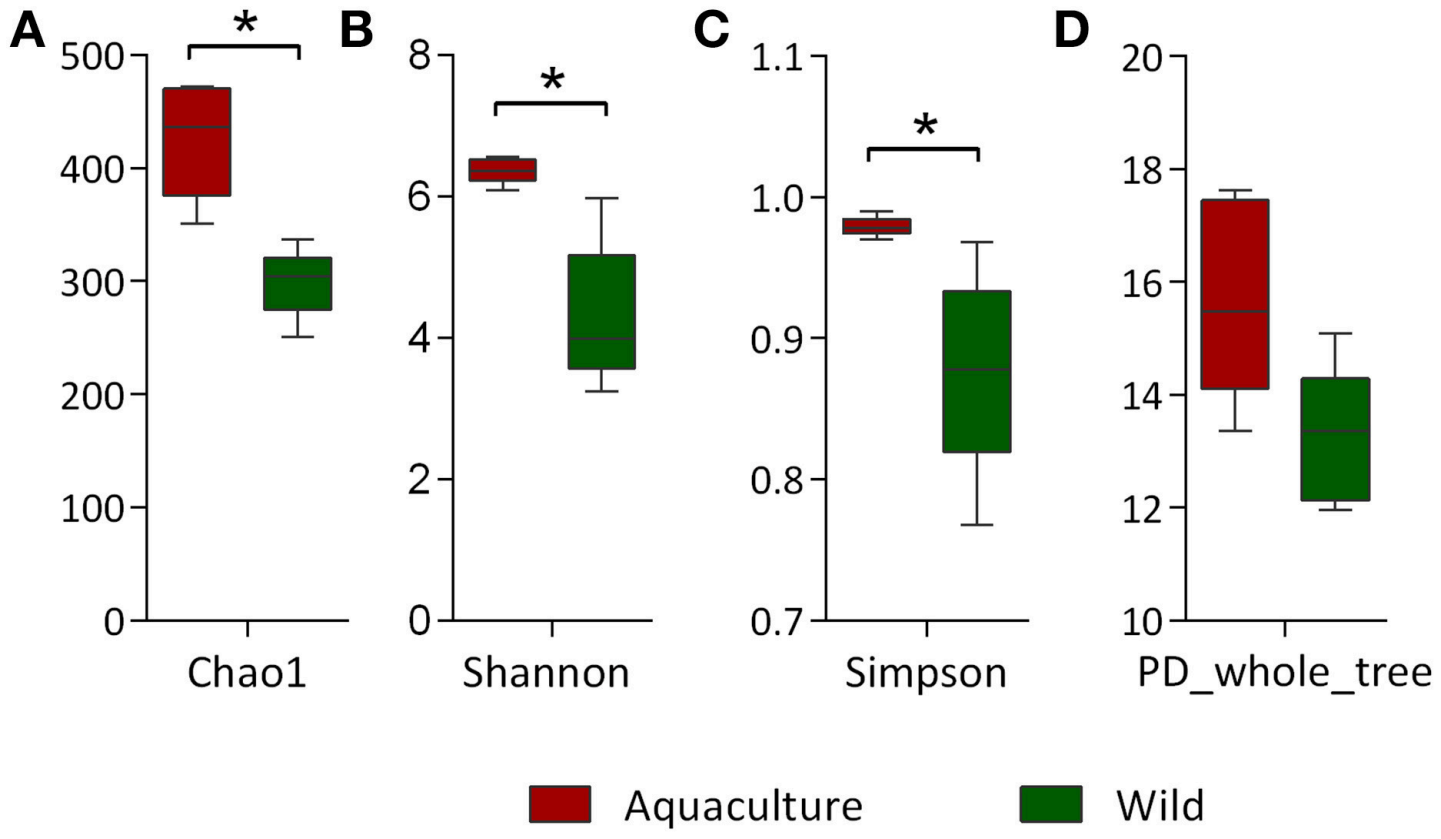
Comparison of alpha diversity indexes between wild and aquaculture yellowtail kingfish (*Seriola lalandi*). Diversity in the gut bacterial community was measured using Chao-1 **(A)**, Shannon index **(B)**, Simpson index **(C)**, and phylogeny-based metrics **(D)**. The asterisks indicate significant differences in the alpha diversity between the wild and aquaculture yellowtail (*P* < 0.05). Chao-1 and PD whole tree was evaluated using *t*-test. Shannon and Simpson index were evaluated using Mann-Whitney.

**Figure 2:**
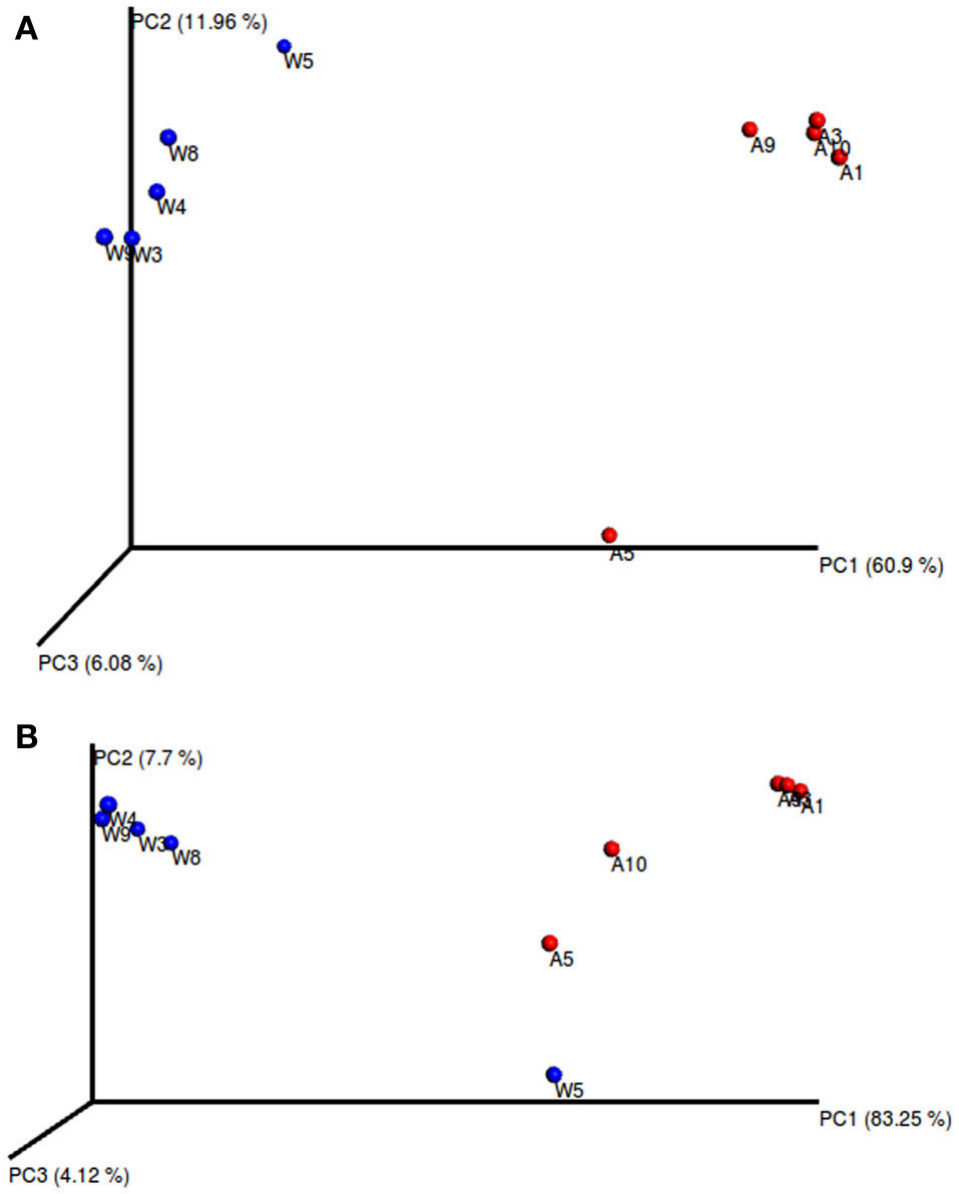
Principal coordinates analysis (PCoA) of the bacterial communities derived from the unweighted **(A)** and weigthed **(B)** UniFrac distance matrix. Circles represent individual samples from *S. lalandi* intestinal microbiota. Red circles correspond to samples derived from aquaculture, and blue circles correspond to samples from wild fish.

The alpha diversity indexes, Chao1, Shannon and Simpson, were found to be significantly higher for the microbiota of aquacultured samples (Figure 1). Beta diversity analysis are illustrated as principal coordinates plots in Figure 2, showing notable differentiation among bacterial communities, as they grouped by origin (wild or aquacultured animals). The graphical representation for unweighted UniFrac analyses highlights the variability in the microbiota composition between wild and aquacultures fish (Figure 2A). Furthermore, PERMANOVA and ANOSIM tests (*P* < 0.05) confirmed the significant differences in the composition of the microbiota depending on the origin of the fish (Table 1).

**Table 1:** Comparison of similarities in microbiota composition between wild and aquaculture yellowtail kingfish.

	**Statistical Test**	**Test Statistic**	***P*-value**
Unweighted UniFrac	PERMANOVA	11.37	0.0072^*^
	ANOSIM	1.00	0.0088^*^
Weighted UniFrac	PERMANOVA	14.19	0.0076^*^
	ANOSIM	0.78	0.0156^*^

* *Indicates a rejection of the null hypothesis of no differences among groups (P < 0.05)*.

### Composition of microbial communities

**Figure 3:**
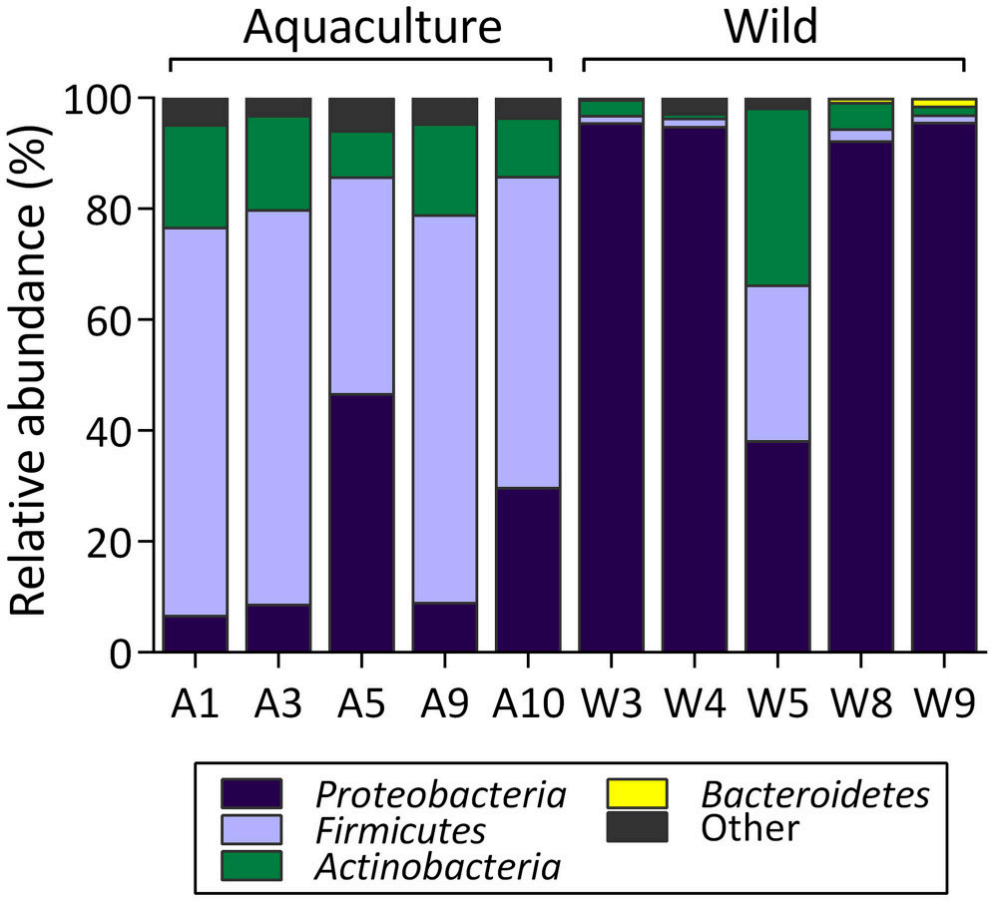
Relative abundance (percentage) at phylum level for each sample in the intestinal microbiota from wild and aquaculture *S. lalandi*. In the figure, A corresponds to aquaculture fish (Aquaculture *S. lalandi*) and W corresponds to individual wild fish (Wild *S. lalandi*).

The taxonomic compositions of the *S. lalandi* intestinal microbiota of wild and aquacultured origin primarily present representatives of the phyla *Proteobacteria, Firmicutes*, and *Actinobacteria* followed by *Bacteroidetes, Planctomycetes, Chloroflexi*, and *Cyanobacteria* with low relative abundance (Figure 3). However, differences in relative abundances of each of these phyla were observed depending on the wild or aquacultured origin of the samples. In wild yellowtail, the most abundant phyla were *Proteobacteria* (83%), *Actinobacteria* (8%), and *Firmicutes* (7%). In aquacultured yellowtail, the most abundant phyla were *Firmicutes* (61%), *Proteobacteria* (20%), and *Actinobacteria* (14%). At the class level, the wild yellowtail microbiota consisted of *Gammaproteobacteria* (80%), *Actinobacteria* (8%), *Bacilli* (7%), and *Alphaproteobacteria* (2%) followed by the *Betaproteobacteria* and *Clostridia* in low relative abundance; whereas aquacultured yellowtail presented *Bacilli* (54%), *Actinobacteria* (14%), *Gammaproteobacteria* (11%), *Clostridia* (7%), *Alphaproteobacteria* (5%), and *Betaproteobacteria* (3%). At the order level, the following bacteria were observed in the wild yellowtail samples: *Pseudomonadales* (52%), *Alteromonadales* (23%), *Actinomycetales* (8%), *Vibrionales* (5%), *Bacillales* (3%), and *Lactobacillales* (3%); whereas aquacultured yellowtail presented *Bacillales* (34%), *Lactobacillales* (20%), *Actinomycetales* (14%), *Pseudomonadales* (10%), *Clostridiales* (6%), and *Sphingomonadales* (4%) (Table S2).

### Differential abundances of the bacterial populations associated with origin

**Figure 4:**
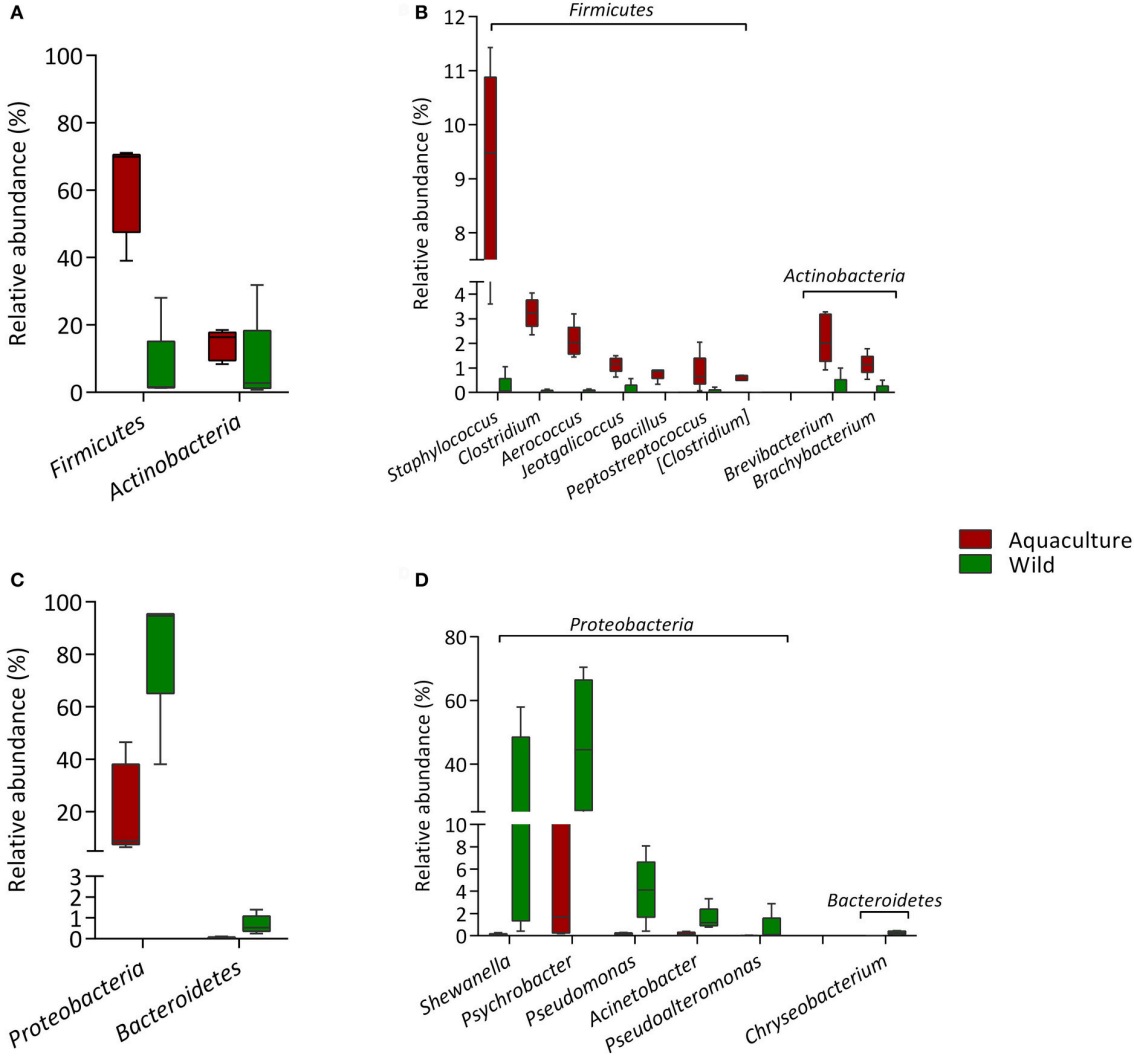
Comparison of the intestinal microbiota between wild and aquaculture yellowtail. This figure illustrates the differences in the microbiota in terms of relative abundance of taxa (Phylum; genus). **(A)** This section highlights the most abundant phyla in aquaculture yellowtail kingfish; **(B)** this shows most abundant genera in aquaculture yellowtail; **(C)** this section highlights the most abundant phyla in wild yellowtail kingfish; **(D)** this shows most abundant genera in wild yellowtail. Only statistical significant taxa are represented, according to DESeq2 analysis.

Specific taxa that were differentially distributed between the wild and aquacultured yellowtail were identified using DESeq2 software package. This approach allows significant differences in the abundances of each OTU to be identified based on statistical tools. The results are shown in Table 2, which depicts each phylum and genus presenting a significant difference between the two origins, aquaculture (positive) and wild (negative). In order to visualize these differences, Figure 4 was structured using the relative abundance of these OTUs as taxa. Figure 4A shows the bacterial components are more abundant in aquacultured yellowtail, starting at the phylum level, in this case corresponding to *Firmicutes* and *Actinobacteria*. In more detail, Figure 4B shows the differences at the genus level within those phyla, highlighting *Staphylococcus, Clostridium, Aerococcus, Jeotgalicoccus*. In contrast, in wild yellowtail, *Proteobacteria* was the most abundant phyla as shown in Figure 4C, which highlights the abundance of *Shewanella, Psychrobacter*, and *Pseudomonas* (Figure 4D).

**Table 2:** Summary of differential abundance at phylum and genus level between wild and aquaculture yellowtail kingfish.

**Phylum**	**Family**	**Genus**	**Log 2 fold difference^a^**	***P* adjusted^b^**
*Firmicutes*			4.1801	<0.001
	*Peptostreptococcaceae*	*Clostridium*	4.5247	<0.001
	*Clostridiaceae*	*Clostridium*	3.5085	<0.001
	*Bacillaceae*	*Bacillus*	4.8228	<0.001
	*Aerococcaceae*	*Aerococcus*	2.4920	<0.001
	*Staphylococcaceae*	*Staphylococcus*	2.9428	<0.001
	*Peptostreptococcaceae*	*Peptostreptococcus*	2.5490	0.004
	*Staphylococcaceae*	*Jeotgalicoccus*	1.4133	0.034
*Actinobacteria*			1.8138	<0.001
	*Brevibacteriaceae*	*Brevibacterium*	1.4874	0.025
	*Dermabacteraceae*	*Brachybacterium*	1.2782	0.032
*Proteobacteria*			−2.2964	<0.001
	*Shewanellaceae*	*Shewanella*	−12.519	<0.001
	*Pseudomonadaceae*	*Pseudomonas*	−9.006	<0.001
	*Moraxellaceae*	*Acinetobacter*	−7.469	<0.001
	*Pseudoalteromonadaceae*	*Pseudoalteromonas*	−9.376	<0.001
	*Moraxellaceae*	*Psychrobacter*	−5.997	<0.001
*Bacteroidetes*			−3.8458	<0.001
	*Flavobacteriaceae*	*Chryseobacterium*	−10.803	<0.001

a *Analysis done using DESeq2 package. Positive values indicate a higher abundance in aquaculture yellowtail compared to wild yellowtail. Negative values indicate a lower abundance in aquaculture yellowtail compared to wild yellowtail*.

b *Adjusted P-value; accounts for multiple testing and controls the false discovery rate*.

### Potential functions of yellowtail metagenome showing significant differences

**Figure 5:**
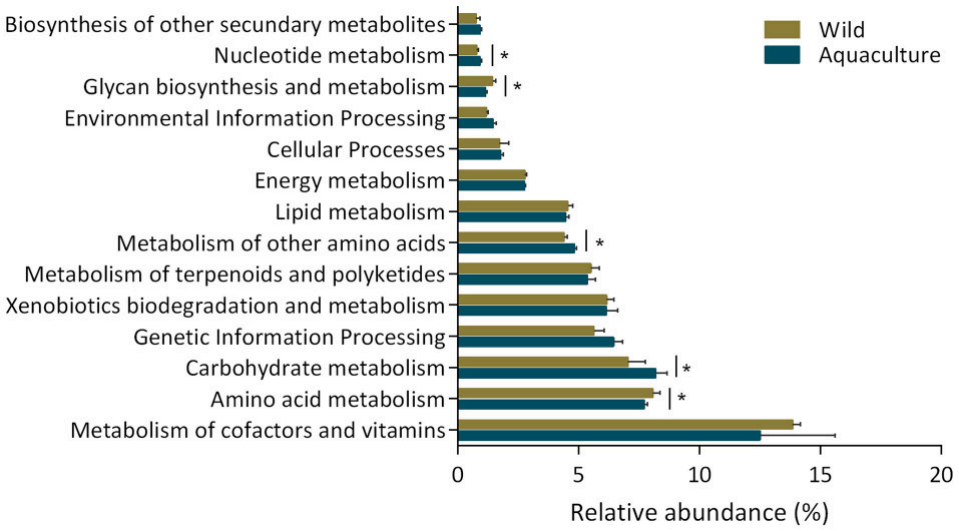
The general metabolic pathways of the intestinal microbiota from wild and aquaculture *S. lalandi*. The asterisks indicate significant differences in pathways of the bacterial components between wild and aquaculture yellowtail kingfish, this was assessed using *t*-test, *P*-values were corrected with the Benjamini–Hochberg false discovery rate method. Those values were considered significant *P* < 0.05.

The changes in the presumptive functions of the intestinal microbiota of yellowtail were examined by predicting the metagenomes using PICRUSt. The accuracy of the prediction was evaluated by computing the Nearest Sequenced Taxon Index (NSTI), and the mean of the samples was 0.048 ± 0.005, indicating a relatively good match to reference genomes (ideal NSTI ≤ 0.03; Langille et al., 2013). Figure 5 depicts the general metabolic pathways, comparing microbiota functions from animals of both origins, which highlights the significant differential distribution of pathways, including those related to amino acid metabolism, carbohydrate metabolism and nucleotide metabolism. Furthermore, 19 functional pathways were found to be more highly abundant in wild yellowtail, including pathways related to the biodegradation of xenobiotics, and the metabolism of terpenoids and polyketides. In the case of aquacultured yellowtail, 31 pathways were found to be more highly abundant, including those related to amino acid metabolism, carbohydrate metabolism, and nucleotide metabolism. The significant differences in bacterial function between wild and aquacultured yellowtail is detailed in Table 3.

**Table 3:** Summary of differences in pathways between predicted metagenomes.

**KEGG pathways**	**Wild**	**Aquaculture**	**FDR**
**Metabolism of cofactors and vitamins**			
Nicotinate and nicotinamide metabolism	**0.61 ± 0.05**	0.53 ± 0.03	0.0083
Riboflavin metabolism	**0.60 ± 0.03**	0.53 ± 0.02	0.0017
Vitamin B6 metabolism	**0.55 ± 0.03**	0.50 ± 0.01	0.0073
Ubiquinone and other terpenoid quinone biosynthesis	**0.43 ± 0.02**	0.36 ± 0.01	0.0004
Thiamine metabolism	0.64 ± 0.08	**0.88 ± 0.09**	0.0019
**Amino acid metabolism**			
Tryptophan metabolism	**0.61 ± 0.04**	0.48 ± 0.07	0.0060
Lysine degradation	**0.60 ± 0.06**	0.45 ± 0.08	0.0076
Phenylalanine tyrosine and tryptophan biosynthesis	**0.51 ± 0.02**	0.49 ± 0.01	0.0100
Lysine biosynthesis	0.66 ± 0.03	**0.73 ± 0.03**	0.0033
**Carbohydrate metabolism**			
Pentose phosphate pathway	0.48 ± 0.07	**0.67 ± 0.1**	0.0079
Fructose and mannose metabolism	0.26 ± 0.09	**0.50 ± 0.1**	0.0044
Amino sugar and nucleotide sugar metabolism	0.34 ± 0.05	**0.49 ± 0.07**	0.0057
Pentose and glucuronate interconversions	0.26 ± 0.05	**0.35 ± 0.03**	0.0051
Galactose metabolism	0.17 ± 0.06	**0.35 ± 0.08**	0.0050
**Genetic information processing**			
Mismatch repair	0.58 ± 0.03	**0.69 ± 0.04**	0.0021
DNA replication	0.45 ± 0.04	**0.54 ± 0.03**	0.0041
RNA polymerase	0.28 ± 0.03	**0.37 ± 0.03**	0.0005
**Xenobiotics biodegradation and metabolism**			
Caprolactam degradation	**1.26 ± 0.19**	0.73 ± 0.26	0.0060
Toluene degradation	**0.38 ± 0.03**	0.31 ± 0.03	0.0038
Styrene degradation	**0.32 ± 0.04**	0.19 ± 0.05	0.0011
Nitrotoluene degradation	**0.12 ± 0.019**	0.08 ± 0.02	0.0066
Chloroalkane and chloroalkene degradation	0.46 ± 0.035	**0.55 ± 0.02**	0.0008
Bisphenol degradation	0.33 ± 0.05	**0.46 ± 0.01**	0.0006
Ethylbenzene degradation	0.30 ± 0.04	**0.42 ± 0.01**	<0.0001
Dioxin degradation	0.07 ± 0.03	**0.23 ± 0.04**	0.0001
Xylene degradation	0.05 ± 0.02	**0.12 ± 0.02**	0.0005
**Metabolism of terpenoids and polyketides**			
Geraniol degradation	**1.58 ± 0.25**	0.94 ± 0.31	0.0075
Biosynthesis of type II polyketide products	0.002 ± 0.0	**0.03 ± 0.02**	0.0033
Carotenoid biosynthesis	0.02 ± 0.02	**0.05 ± 0.01**	0.0059
Biosynthesis of ansamycins	0.84 ± 0.12	**1.14 ± 0.07**	0.0013
**Metabolism of other amino acids**			
Beta Alanine metabolism	**0.88 ± 0.08**	0.66 ± 0.12	0.0096
Glutathione metabolism	**0.67 ± 0.08**	0.47 ± 0.09	0.0061
D Alanine metabolism	0.58 ± 0.05	**0.79 ± 009**	0.0016
D Arginine and D ornithine metabolism	0.06 ± 0.08	**0.33 ± 0.07**	0.0004
**Lipid metabolism**			
Biosynthesis of unsaturated fatty acids	**0.65 ± 0.11**	0.39 ± 0.08	0.0027
Fatty acid biosynthesis	0.92 ± 0.02	**1.02 ± 0.05**	0.0019
Glycerolipid metabolism	0.25 ± 0.03	**0.32 ± 0.03**	0.0044
Glycerophospholipid metabolism	0.30 ± 0.0	**0.32 ± 0.01**	0.0038
Secondary bile acid biosynthesis	0.06 ± 0.04	**0.28 ± 0.08**	0.0005
Linoleic acid metabolism	0.17 ± 0.04	**0.24 ± 0.01**	0.0052
Sphingolipid metabolism	0.02 ± 0.01	**0.05 ± 0.01**	0.0081
**Energy metabolism**			
Carbon fixation pathways in prokaryotes	**0.77 ± 0.05**	0.65 ± 0.05	0.0061
Nitrogen metabolism	**0.37 ± 0.02**	0.34 ± 0.01	0.0030
Carbon fixation in photosynthetic organisms	0.49 ± 0.03	**0.58 ± 0.03**	0.0031
Photosynthesis	0.19 ± 0.01	**0.22 ± 0.01**	0.0043

*The values in bold correspond to significantly greater abundances with respect to the condition with which it was compared*.

## Discussion

To our knowledge, this is the first study to characterize and compare the intestinal microbiota of wild and aquacultured *S. lalandi* using high-throughput sequencing. A previous study by Aguilera et al. (2013) examined the culture dependent microbiota of yellowtail juveniles in an aquaculture system. The authors described that in an aquaculture system the microbiota was represented by the phylum *Proteobacteria*. These results contrast with those obtained in our study, where the intestinal microbiota of aquacultured yellowtail was dominated by the phylum *Firmicutes*, exhibiting 61% of relative the abundance, whereas the phylum *Proteobacteria* only reached a 20% relative abundance (Figure 4A). The differences of our results with respect to Aguilera et al. (2013) could be attributed to the methodological differences since the microbiota composition obtained using culture-dependent approaches is highly influenced by the culture medium used to perform the isolation of microorganisms. In contrast, culture-independent approaches, such as NGS, exceed the limits of bacterial recovery from the culture medium, which in marine environments does not exceed 1% of cultivable bacteria (Amann et al., 1995). In addition, NGS strategies allow for a more complete view of the composition of the microbiota, with high and deep coverage, allowing the taxonomic classification of bacteria using thousands of reads (Whiteley et al., 2012). This approach has improved our view of the fish microbiome and several reviews have been published recently (Llewellyn et al., 2014; Ghanbari et al., 2015; Tarnecki et al., 2017). These compilations indicate that structure of fish microbiomes could be more similar to the microbiome of their environments than those of mammals; in fact, previous reports addressing gut microbiota in fish have showed that gut microbiota composition was different from those found in the surrounding environment such as feed or water (Kormas et al., 2014; Bakke et al., 2015; Estruch et al., 2015; Li et al., 2015). Therefore, fish microbiome structure are not simply a reflection of the environment, but are a result of both phylogenetic factors and host ecology (Ghanbari et al., 2015).

Our results indicate that wild *S. lalandi* has a different microbiota compared to the aquaculture animals (Figure 2, Table 1). These results could be associated with differences in fish feeding depending on their origin. Gajardo et al. (2017) reported that there are significant changes in beta diversity statistics associated with the *Salmo salar* intestinal microbiota when fed with different diets. Similar to results were described by Schmidt et al. (2016), who suggested that the microbiota compositions were modulated by the diets under a RAS environment, using an ANOSIM test. These authors indicated that the salmon microbiota fed with two diets (fishmeal-based and fishmeal-free) were dominated by *Lactobacillales, Aeromonadales*, and *Actinomycetales*, followed by *Sphingomonadales* and *Clostridiales*; but at the genus level, the microbiota showed significant differences depending on the diet. Similar results were described in *Sparus aurata* (Estruch et al., 2015), where total fishmeal replacement had an important impact on microbial profiles with *Streptococcus* was highly represented in fish fed with fishmeal diets and microbial composition of the RAS was totally different to that of the sea bream gastrointestinal tract. These match with our results where the *S. lalandi* microbiota of aquacultured fish under RAS was dominated by bacteria of the order *Bacilales*.

The differences in relative abundances of the components of the intestinal microbiota of *S. lalandi*, shown in Figure 4, indicate that in the case of wild yellowtail the predominant phylum is *Proteobacteria*. This phylum has been described as the most predominant in freshwater wild fish by Liu et al. (2016), as well as in wild marine fish by Star et al. (2013) and Ramírez and Romero (2017). In those reports, the most abundant class identified within this phylum was *Gammaproteobacteria*, similar to the results of our study. The order *Pseudomonadales* was the most abundant and contained differentially represented genera in wild yellowtail, such as *Psychrobacter, Pseudomonas*, and *Acinetobacter*.

*Psychrobacter* sp. has been described in the intestinal microbiota of several fish, such as Atlantic cod (Ringø et al., 2006), grouper (Sun et al., 2009; Yang et al., 2011) and fine flounder (Ramírez and Romero, 2017). *Psychrobacter* sp. has previously showed an *in vitro* antagonistic effect against a number of pathogenic species, such as *S. aureus, Vibrio harveyi, Vibrio metschnikovii*, and *Vibrio alginolyticus* (Sun et al., 2009). This may be an important observation because vibriosis has been reported as a primary disease in hatcheries and cultures of marine fish (Reid et al., 2009). More recently, Makled et al. (2017) reported that the dietary administration of a strain of *Psychrobacter* improved immunological parameters and growth performance in tilapia. Similarly, *Pseudomonas* is a very diverse group showing adaptability to a range of environmental niches and a broad ecological distribution. *Pseudomonas* strains have a wide variety of metabolic abilities and some of them have been used as probiotics in aquaculture, improving the response of different hosts to pathogens (Alavandi et al., 2004; Korkea-aho et al., 2011; Giri et al., 2014). In contrast, Acinetobacter has been reported in the microbiota of several fish, such as rainbow trout (Spanggaard et al., 2001), Atlantic salmon (Navarrete et al., 2009) and yellowtail kingfish (Aguilera et al., 2013). This bacterial genus has been reported to inhabit soil and aquatic environments, including freshwater ecosystems, raw sewage and wastewater treatment plants, as well as activated sludge (Doughari et al., 2011). They are primarily free-living saprophytes that are found ubiquitously in nature and have a variety of metabolic capabilities, such as the degradation of aromatic compounds (Mazzoli et al., 2007). Another genus with significant relative abundance in wild yellowtail was *Shewanella*; several strains of this genus have previously been reported to produce polyunsaturated fatty acids (Hirota et al., 2005; Bianchi et al., 2014) including some strains retrieved from microbiota of freshwater fish Dailey et al. (2016). *Shewanella* strains have shown probiotic properties, improving survival against vibriosis in *Solea senegalensis* (Tapia-Paniagua et al., 2014) and enhancing immune parameters in *Sparus aurata* L (Cordero et al., 2015).

In contrast to wild yellowtail, the statistically dominant phylum in aquaculture specimens was *Firmicutes* (Figure 4A). This phylum has been described as the most predominant in the intestinal content of aquacultured fishes such as Siberian sturgeon (Geraylou et al., 2013), grass carp (Wu et al., 2012) and Atlantic salmon (Schmidt et al., 2016). At the genus level, *Staphylococcus, Clostridium, Aerococcus, Brevibacterium*, were the most abundant (Figure 4C). Bacteria of these genera have been described as beneficial bacteria for hosts, such as *Brevibacterium* spp., and *Staphylococcus* spp., and they may contribute to nutritional processes in Arctic charr (Ringø et al., 1995). Furthermore, oral administration of *Clostridium butyricum* to rainbow trout enhanced their resistance to vibriosis by increasing the phagocytic activity of leucocytes (Sakai et al., 1995). Some strains of *Aerococcus* have also been described as an antagonist to opportunist pathogens (Burbank et al., 2012; Valchuk et al., 2015).

The PICRUSt analysis was used to infer functional capabilities of the microbial communities. This approach predicts functional potentials of a community by comparing its metagenome with reference genomes (Langille et al., 2013). According to metagenome prediction, we inferred that the most abundant functional categories were those corresponding to the functions of the metabolism of cofactors and vitamins, amino acid metabolism, and carbohydrate metabolism (Figure 5) and found significant differences in KEGG pathways between wild and aquacultured yellowtail (Table 2). For example, pathways relating to the metabolism of group B vitamins and ubiquinone biosynthesis were more abundant in the wild yellowtail microbiota. Ubiquinone has been described as having bioactivities related to energy metabolism, immunological competence, and antioxidation (Pravst et al., 2010). Similarly, KEGG pathways related to amino acid metabolism, such as tryptophan and phenylalanine, were significantly associated with wild yellowtail, whereas lysine biosynthesis was associated with aquacultured fish. These amino acids are commonly mentioned as essential nutritional requirements in several fish (Wilson and Halver, 1986). In terms of carbohydrate metabolism, the pentose and galactose pathways were more significantly represented in aquacultured yellowtail. This observation could be related to the artificial diet (Table S4), because it contains a larger amount of carbohydrates (>12%) with respect to common prey for wild yellowtail, such as fish, shrimp, and squid (< 6%; Sidwell et al., 1974). In contrast, lipopolysaccharide biosynthesis was significantly associated with wild yellowtail, and this is coincident with a larger abundance of *Proteobacteria* in these fish, which are gram-negative bacteria harboring LPS genes. Another important KEGG pathway significantly present in wild yellowtail was the biosynthesis of unsaturated fatty acids, which include essential omega-3 fatty acids (eicosapentaenoic acid and docosahexaenoic acid). As mentioned before, *Gammaproteobacteria* was dominant in wild fish, and this class includes the previously reported EPA/DHA bacterial producers (Dailey et al., 2016; Ramírez and Romero, 2017). However, PICRUSt is a predictor of potential functions within a metagenome; metabolomic approaches could be used to identify differences in the metabolic functions in new environments such as the microbiota of wild and aquaculture fish (Gajardo et al., 2016).

In conclusion, this study reveals the differences in the composition of the intestinal microbiota of wild origin and aquacultured *S. lalandi*. These differences likely result in different potential contributions of the microbiota to the host. These results indicate a strong influence of host feeding on the composition and diversity of the intestinal microbiota. The bacterial genera differentially represented between fish of both origins present positive characteristics for the host, especially those associated with wild yellowtail. Therefore, it would be interesting to evaluate the activity of isolates of these genera as potential probiotics for their use in *S. lalandi* aquaculture. Our findings could provide a promising direction for the healthy aquaculture of *S. lalandi*.

## Author contributions

CR Performed DNA extraction; data collection; data analysis, and interpretation; wrote the initial version of the manuscript. JR Conception or design of the work; contributed to the discussion and writing.

### Conflict of interest statement

The authors declare that the research was conducted in the absence of any commercial or financial relationships that could be construed as a potential conflict of interest.
