# Supplementary material for: The Microbiome of Seriola lalandi of Wild and Aquaculture Origin Reveals Differences in Composition and Potential Function
Source: Front Microbiol. 2017 Sep 26;8:1844. doi: 10.3389/fmicb.2017.01844 (PMC5622978; doi:10.3389/fmicb.2017.01844)
Supplement: Supplementary file 2 [file Presentation1.PPTX]

## Slide 1
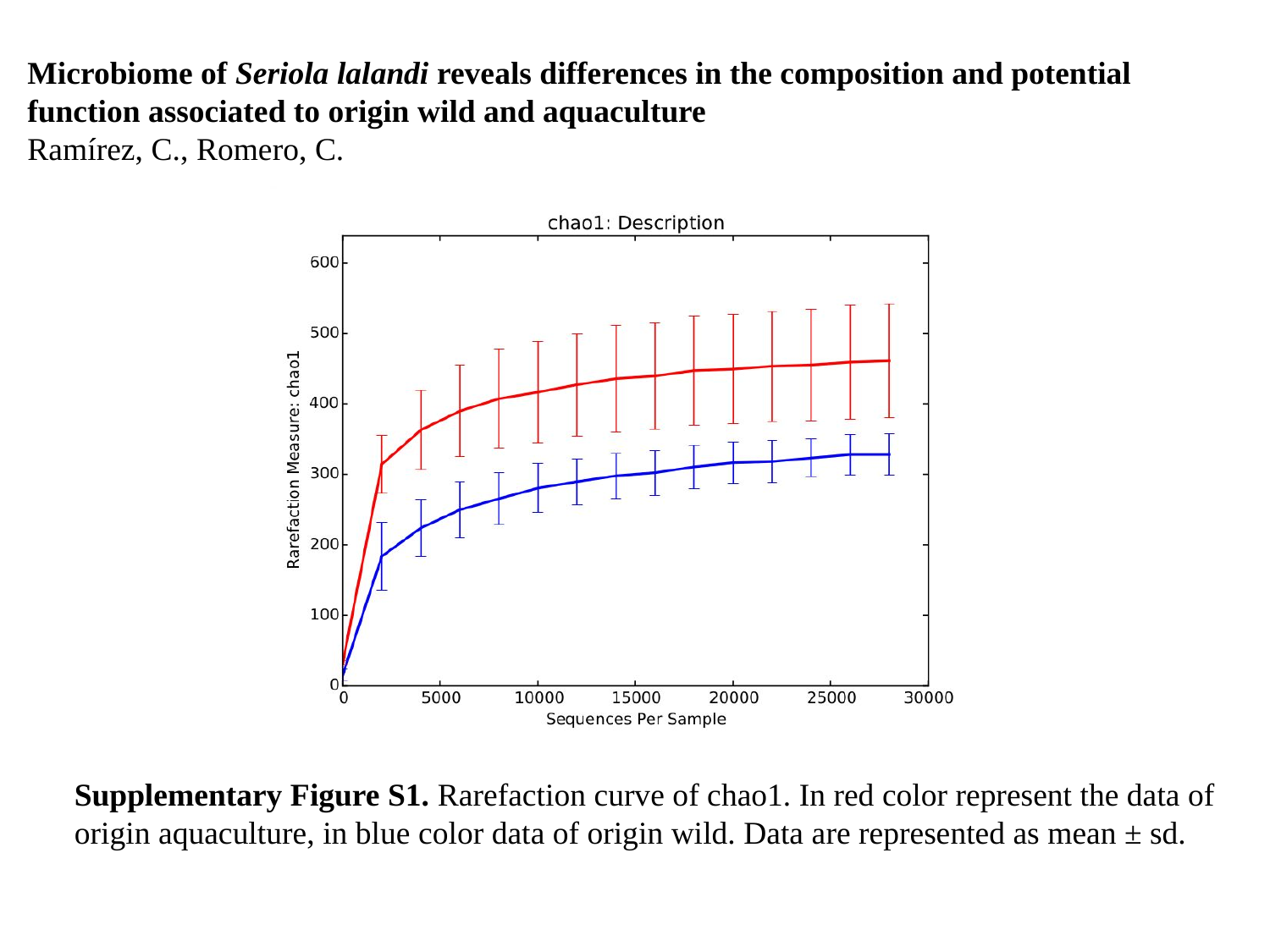

Microbiome of Seriola lalandi reveals differences in the composition and potential function associated to origin wild and aquaculture
Ramírez, C., Romero, C.
Supplementary Figure S1. Rarefaction curve of chao1. In red color represent the data of origin aquaculture, in blue color data of origin wild. Data are represented as mean ± sd.

## Slide 2
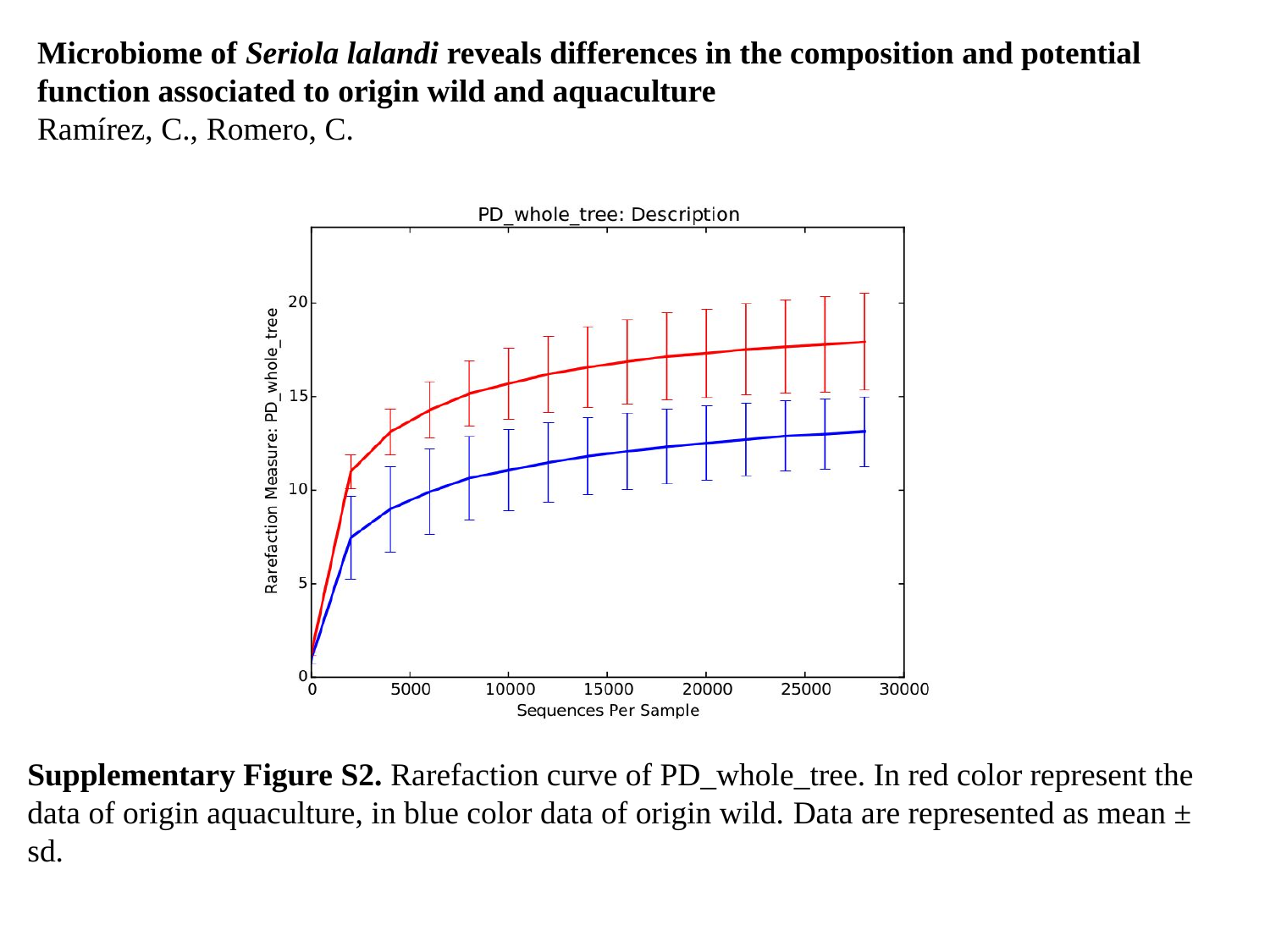

Microbiome of Seriola lalandi reveals differences in the composition and potential function associated to origin wild and aquaculture
Ramírez, C., Romero, C.
Supplementary Figure S2. Rarefaction curve of PD_whole_tree. In red color represent the data of origin aquaculture, in blue color data of origin wild. Data are represented as mean ± sd.
